# The Medical and Dental Laws

**Published:** 1882-01

**Authors:** 


### THE MEDICAL AND DENTAL LAWS.

Judge Avery, in a decision touching the "continuously engaged in practice" clause of the medical law, says: (*Cin. Gazette*, Nov. 1, 1881) "The ten years' practice named in the act could have been intended to refer only to such practice prior to its passage. After the act any such practice would be unlawful; and it could not be that the legislature intended that what was made unlawful should, by continuing, become lawful."

This construction would, it seems to me, apply also to the similar clause in the dental law.

